# Receptor recharge time drastically reduces the number of captured particles

**DOI:** 10.1371/journal.pcbi.1006015

**Published:** 2018-03-01

**Authors:** Gregory Handy, Sean D. Lawley, Alla Borisyuk

**Affiliations:** Department of Mathematics, University of Utah, Salt Lake City, Utah, United States of America; University of Pittsburgh, UNITED STATES

## Abstract

Many diverse biological systems are described by randomly moving particles that can be captured by traps in their environment. Examples include neurotransmitters diffusing in the synaptic cleft before binding to receptors and prey roaming an environment before capture by predators. In most cases, the traps cannot capture particles continuously. Rather, each trap must wait a transitory “recharge” time after capturing a particle before additional captures. This recharge time is often overlooked. In the case of instant recharge, the average number of particles captured before they escape grows linearly in the total number of particles. In stark contrast, we prove that for any nonzero recharge time, the average number of captured particles grows at most logarithmically in the total particle number. This is a fundamental effect of recharge, as it holds under very general assumptions on particle motion and spatial domain. Furthermore, we characterize the parameter regime in which a given recharge time will dramatically affect a system, allowing researchers to easily verify if they need to account for recharge in their specific system. Finally, we consider a few examples, including a neural system in which recharge reduces neurotransmitter bindings by several orders of magnitude.

## Author summary

Consider particles that are released into an environment (think diffusing molecules or plankton), and suppose that there are traps in the environment. How many particles will be captured by the traps before they escape? In a standard model, the number of captured particles is proportional to the initial number released. In this paper, we show that for a more realistic model of a trap (one in which traps must recharge after every capture), the number of captures is proportional to the logarithm of the initial number released. That means that if 10^6^ particles are released, only about 6 will be captured. We prove this result mathematically, and then consider a number of applications, including neuronal synapses and ambush predators.

## Introduction

Particles moving and interacting with traps is a broad description of many biological processes. In individual applications, “particles” might represent, for example, molecules or prey, while “traps” could represent receptors or predators. Nevertheless, the mathematical description can be very similar.

**Fig 1:**
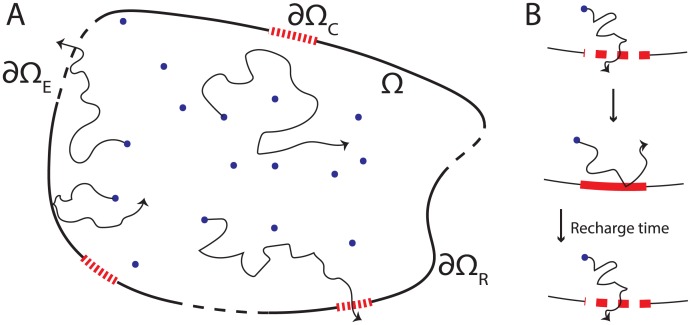
Schematics of domain and recharging capture regions. A: Particles diffusing in domain Ω with boundary ∂Ω = ∂Ω_*R*_ ∪ ∂Ω_*C*_ ∪ ∂Ω_*E*_. B: After capturing a particle, capture regions are reflecting for a transitory recharge time, which we take to be exponentially distributed with mean *τ*_*r*_ > 0.

In this work we consider a finite number of particles randomly moving in a bounded domain. Eventually, each particle will leave the domain through either an *escape* region in the boundary or a *capture* region in the boundary (Fig 1). After a capture region captures a particle, that region cannot capture additional particles until after a transitory *recharge* time. We find that this recharge time can dramatically reduce the number of particles that are captured before they escape.

One motivation for this study is the interaction of neurotransmitter with receptors in the synaptic cleft. The synaptic cleft is a small region in extracellular space between neuronal processes [1]. Once a neuron activates, it releases a packet of neurotransmitter molecules (“particles”) into the cleft, where they diffuse until they either leave the cleft (escape) or bind to the synaptic receptors on the membrane of the other neuron (are captured). The receptor that captures a molecule changes conformation, and during this time it cannot capture additional molecules. After a transitory recharge time, the receptor returns to its original state in which it can capture molecules. A similar scenario occurs, for example, in experiments where the molecules are released into extracellular space and bind to receptors on astrocytes, another type of brain cell [2].

In an application to ecology, the capture regions could represent ambush predators [3]. These are organisms that stay stationary, while the prey (“particles”) wander about. Once prey is within a striking range, the predator attacks and captures the prey. The recharge in this case represents the so-called handling time [4, 5], which is the time spent processing food by the predator, until it is ready to hunt again. Examples of such predators can be found in different taxa, including carnivorous plants, chameleons, some fish, and spiders.

Here we consider *n* non-interacting particles randomly moving in a bounded domain $\Omega \subset R^{d}$ in any spatial dimension, *d* ≥ 1. For simplicity, we assume the particles are purely diffusing, but our results hold under much more general assumptions (see the Discussion). The boundary ∂Ω is partitioned into *reflecting* regions ∂Ω_*R*_ which reflect particles, *escape* regions ∂Ω_*E*_ which absorb particles, and *m*-many *capture* regions $\partial \Omega_{C}=\cup_{k=1}^{m}\partial \Omega_{C}^{j}$, see Fig 1A. Each capture region $\partial \Omega_{C}^{j}$ absorbs particles, except during a transitory time after it absorbs a particle in which the region is reflecting, see Fig 1B. We take this transitory time to be exponentially distributed with mean *τ*_*r*_ > 0 and envision it as the time required for a capture region to “recharge” before it can capture another particle.

Eventually, each particle will either be absorbed at an escape region (in which case we say the particle has *escaped*), or absorbed at a capture region (in which case we say the particle was *captured*). Let *N* ≥ 0 be the number of particles that are eventually captured. In the case of instant recharge (*τ*_*r*_ = 0) and independently moving particles, the expected value of *N* is simply

$$\begin{array}{c} E[N]=hn, \end{array}$$ (1)

where *h* ∈ [0, 1] is the probability that a given particle reaches a capture region before an escape region (the so-called hitting probability).

In this paper, we investigate the effect of a nonzero recharge time *τ*_*r*_ > 0 on the expected number of captured particles, E[N]. Notice that particles still move independently, but they now interact through their effect on the state of the boundary. In contrast to the linear growth of E[N] as a function of *n* in Eq 1 in the case *τ*_*r*_ = 0, we prove that if *τ*_*r*_ > 0, then E[N] cannot grow faster than logarithmically for large *n*. We then demonstrate through numerical simulations that E[N] does indeed grow logarithmically (rather than sublogarithmically). Furthermore, we characterize this growth in terms of only three biological parameters. Namely, if *n* is the number of particles released into the domain, *m* is the number of capture regions, and *T* = *τ*_*r*_/*τ*_*e*_ is the ratio of the expected recharge time *τ*_*r*_ to the expected escape time *τ*_*e*_ if all the capture regions are always reflecting, then the upper bound for E[N] is approximately equal to

$$\begin{array}{c} m+\frac{m}{T}(\log(n\frac{T}{m})+1). \end{array}$$ (2)

We make Eq 2 precise in Theorems 1 and 2 below. In addition, we provide in Eq 16 a simple condition to check if a particular parameter regime is such that E[N] is dramatically reduced by the recharge time.

## Materials and methods

**Fig 2:**
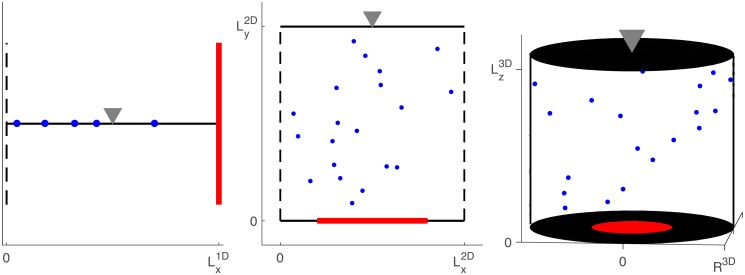
Three example domains. A: Ω^1D^, B: Ω^2D^, C: Ω^3D^, with escape regions denoted by black, dashed lines, capture regions by red, solid lines, and reflecting regions by black, solid lines. Unless otherwise specified, all particles are initially located at the gray triangles in each domain.

We compare the upper bound presented in Theorem 2 to simulations conducted in the three domains illustrated in Fig 2. In these domains, the PDE in Eq 11 can be solved using separation of variables. With this solution, it is straightforward to estimate the constant *C* using Eq 12. The results of this calculation, assuming the initial distribution of particles is given by a delta-distribution, can be found in Table 1. Unless specified otherwise, all simulations used these values for λ_1_ and *C*. Parameter values for *n*, *τ*, *D*, $L_{x}^{\text{1D}}$, $L_{x}^{\text{2D}}$, $L_{y}^{\text{2D}}$, $L_{z}^{\text{3D}}$, and *R*^3D^ can be found in the figure captions, along with the locations of the capture regions for Ω^2D^ and Ω^3D^.

**Table 1:** Eigenvalues (for *k* = 1, 2, …), and coefficients for Eq 12 for the domains found in Fig 2, where the initial distributions of particles are: Ω^1D^: $p\left(x\right)=\delta \left(x-0.5L_{x}^{\text{1D}}\right)$, Ω^2D^: $\delta \left(x,y\right)=\delta \left(x-0.5L_{x}^{\text{2D}}\right)\delta \left(y-\left(L_{y}^{\text{2D}}\right)\right)$, and Ω^3D^: $\delta \left(x,y,z\right)=\delta \left(x\right)\delta \left(y\right)\delta (z-\left(L_{z}^{\text{3D}}\right))$. *J*_0_(*r*) and *J*_1_(*r*) denote the zeroth and first order Bessel function of the first kind, respectively, and *α*_*k*_ denotes the k^th^ zero *J*_0_(*r*).

	λ_*k*_	*A*_*k*_	C
Ω^1D^	${\left(\frac{\pi +2\pi \left(k-1\right)}{2L_{x}^{\text{1D}}}\right)}^{2}$	$\frac{4}{\pi +2\pi k}\sin(\frac{\pi +2\pi (k-1)}{4})$	1.0377
Ω^2D^	${\left(\frac{\pi k}{L_{x}^{\text{2D}}}\right)}^{2}$	$\frac{2(1-{\left(-1\right)}^{k})}{\pi (k+1)}\sin(\frac{\pi k}{2})$	1.2732
Ω^3D^	${\left(\frac{\alpha_{k}}{R^{\text{3D}}}\right)}^{2}$	$\frac{2}{J_{1}\left(\alpha_{k}\right)\alpha_{k}}$	1.6020

The PDE for the probability of hitting a capture region for Ω^2D^ and Ω^3D^ was solved numerically using the NDSolveValue function in Mathematica [6].

For the three-dimensional synaptic cleft, the parameter values $L_{z}^{\text{3D}}$, *R*^3D^, *D* as well as the number of capture regions (*m*) for NMDA and AMPA clefts were found in [7], *n* was found in [8], and the size of the individual capture regions were estimated from [9]. The recharge times (*τ*_*r*_) for AMPA and NMDA receptors were estimated from kinetic schemes from [10] and [11]. Specifically, the recharge times were taken to be the inverse of the unbinding rate of glutamate in the open state of these kinetic schemes. For the predator-prey example, $L_{z}^{\text{3D}}$, *R*^3D^, *n*, *m*, and the handling time (*τ*_*r*_) were estimated from [12]. The ambush predator *Chaoborus Americanus* are typically 1.1 ⋅ 10^4^ *μ*m in length [13], and we considered a reasonable capture region with a radius twice that length. While the prey *Daphnia* are capable of swimming, their motion has been modeled with brownian motion, and we used the effective diffusion coefficient reported in [14]. All of these values are in Table 2.

**Table 2:** Parameter values used in applications (Fig 2C). The domain is cylindrical (Fig 2). The initial distribution of particles/prey is $\delta \left(x,y,z\right)=\delta \left(x\right)\delta \left(y\right)\delta (z-\left(L_{z}^{\text{3D}}\right))$. Columns 2, 3: Neuronal synapse. The receptors are taken to be uniformly distributed in {(*x*, *y*, 0)|*x*^2^ + *y*^2^ < (*R*^3D^)^2^}, and each corresponding capture region has a radius of 0.00625 *μ*m. Columns 4: Ambush predator. The predator is at (0, 0, 0) and has a capture radius of 2.2 ⋅ 10^4^ *μ*m.

Parameter	NMDA	AMPA	Predator
*R*^3D^	0.15 *μ*m	0.15 *μ*m	3.5 ⋅ 10^4^ *μ*m
$L_{z}^{\text{3D}}$	0.02 *μ*m	0.02 *μ*m	5.2 ⋅ 10^4^ *μ*m
*D*	0.3(*μ*m)^2^/ms	0.3(*μ*m)^2^/ms	3.255 ⋅ 10^6^(*μ*m)^2^/s
*m*	20	200	1
*n*	3000	3000	30
*τ*_*r*_	10.917 ms	0.25 ms	8.64 ⋅ 10^3^ s

All simulations were completed in C, with a time step of 0.001*μ*s. The simulations ended when no particles remained in the domain. 100 trials were completed for each parameter set.

## Results

### Mathematical results

We now make Eq 2 precise. The following upper bound follows immediately from Eq 1,

$$\begin{array}{c} E\left[N\right]\leq hn,\;\text{if}\;\tau_{r}\geq 0. \end{array}$$ (3)

To get a tighter upper bound, let *S*(*t*) ∈ [0, 1] be the probability that a given particle has not escaped by time *t* ≥ 0 in the case that all the capture regions are always reflecting. The following lemma bounds E[N] in terms of this survival probability, *S*(*t*).

**Lemma 1.** *For each t* ≥ 0, *we have that*

$$\begin{array}{c} E\left[N\right]\leq m\left(1+t/\tau_{r}\right)+nS\left(t\right). \end{array}$$

*Proof*. Let *N*_*s*,*t*_ be the number of particles captured between time *s* ≥ 0 and time *t* ≥ *s*. With probability one we have that

$$\begin{array}{c} N=N_{0,t}+N_{t,\infty },\;\text{if}\;t\geq 0. \end{array}$$ (4)

We can bound *N*_0,*t*_ by noting that all captures, except the first one, are preceded by a recharge time. Since the recharge time is exponentially distributed with mean *τ*_*r*_ > 0, and since there are *m* capture regions, the expected number of recharges before time *t* is *mt*/*τ*_*r*_. Since the *m* capture regions are initially absorbing, we have

$$\begin{array}{c} E\left[N_{0,t}\right]\leq m\left(1+t/\tau_{r}\right),\;\text{if}\;t\geq 0. \end{array}$$ (5)

In words, the righthand side of Eq 5 is achieved if each of the *m* capture regions captures a particle at time zero and then immediately after each recharge time (1 particle each per average recharge time *τ*_*r*_) up to time *t*.

Next, observe that *N*_*t*,∞_ cannot be greater than the number of particles still in the domain at time *t* ≥ 0. Furthermore, assuming all the capture regions are always reflecting can only increase the number of particles still in the domain at time *t* ≥ 0. If the capture regions are always reflecting, then the expected number of particles remaining in the domain at time *t* ≥ 0 is *nS*(*t*). Therefore,

$$\begin{array}{c} E\left[N_{t,\infty }\right]\leq nS\left(t\right),\;\text{if}\;t\geq 0. \end{array}$$ (6)

In words, the right side of Eq 6 is achieved if all the particles that are still in the domain at time *t* are eventually captured (rather than escape). Taking the expectation of Eq 4 and using Eqs 5 and 6 completes the proof.

If *S*(*t*) decays exponentially, then the following theorem follows quickly from Lemma 1 and Eq 3.

**Theorem 1**. *If* λ > 0 *and C* > 0 *are such that*

$$\begin{array}{c} S\left(t\right)\leq Ce^{-\lambda t},\;for\;all\;\;t\geq 0, \end{array}$$ (7)

*then*

$$\begin{array}{c} E[N]\leq \min\{m+\frac{m}{\lambda \tau_{r}}\log^{+}(Cn\frac{\lambda \tau_{r}}{m})+\min\{nC,\frac{m}{\tau_{r}\lambda }\},\;hn\}, \end{array}$$ (8)

*where* log^+^(*y*) ≔ max{log(*y*), 0}.

*Proof*. Combining Lemma 1 with Eq 7, we have that

$$\begin{array}{c} E\left[N\right]\leq m\left(1+t/\tau_{r}\right)+nCe^{-\lambda t},\;t\geq 0. \end{array}$$ (9)

A simple calculus exercise shows that the value of *t* ≥ 0 that minimizes the upper bound in Eq 9 is

$$\begin{array}{c} t=\frac{1}{\lambda }\log^{+}(Cn\frac{\lambda \tau_{r}}{m})\geq 0. \end{array}$$

Plugging this value of *t* into Eq 9 and using Eq 3 completes the proof.

In order to apply Theorem 1 to the situation described in the Introduction, we need to find λ > 0 and *C* > 0 that satisfy Eq 7. The first step is to use separation of variables to find the survival probability *S*(*t*).

**Lemma 2** *Assume ∂*Ω *is the union of a finite number of disjoint closed Lipschitz surfaces, each surface having finite surface area (a smooth boundary with finite surface area satisfies this assumption). Suppose the initial distribution of each particle is p*(*x*) *and define the shorthand notation*,

$$\begin{array}{c} \left(f,g\right)≔\int_{\Omega }f\left(x\right)g\left(x\right)\;dx. \end{array}$$

*If each particle has diffusivity D* > 0, *then the survival probability is given by*

$$\begin{array}{c} S\left(t\right)=\sum_{k=1}^{\infty }A_{k}e^{-D\lambda_{k}t},\;t>0, \end{array}$$ (10)

*where A*_*k*_ ≔ (*ϕ*_*k*_, 1)(*ϕ*_*k*_, *p*) *and*

$$\begin{array}{c} 0<\lambda_{1}<\lambda_{2}\leq \ldots , \end{array}$$

*is the increasing sequence of eigenvalues satisfying* λ_*k*_ → ∞ *as k* → ∞ *and*

$$\begin{array}{cc} \begin{array}{ccc} -\lambda_{k}\phi_{k} & = & \Delta \phi_{k},\\ \phi_{k} & = & 0,\\ \frac{\partial }{\partial \sigma }\phi_{k} & = & 0, \end{array} & \begin{array}{ccc} x & \in & \Omega ,\\ x & \in & \partial \Omega_{E},\\ x & \in & \partial \Omega \backslash \partial \Omega_{E}, \end{array} \end{array}$$ (11)

*for corresponding eigenfunctions* ${\left\{\phi_{k}\left(x\right)\right\}}_{k=1}^{\infty }$ *which form an orthonormal basis for L*^2^(Ω).

We relegate the proof of Lemma 2 to S1 Text. Using Lemma 2, we can find λ > 0 and *C* > 0 that satisfy Eq 7 and apply Theorem 1 to the situation described in the Introduction. In the following, let *τ*_*e*_ ≔ (*D*λ_1_)^−1^, which we refer to as the *escape time* because it is the average time for a particle to reach an escape region if the capture regions are always reflecting and the particle is initially distributed according to its so-called quasi-stationary distribution, *ϕ*_1_(*x*)/(*ϕ*_1_, 1) ≥ 0 (see S1 Text). Further, let *T* ≔ *τ*_*r*_/*τ*_*e*_ be the relative recharge time.

**Theorem 2.** *Under the assumptions of Lemma 2, we have*

$$\begin{array}{c} E[N]\leq \min\{m+\frac{m}{T}\log^{+}(C\frac{nT}{m})+\min\{nC,\frac{m}{T}\},hn\}, \end{array}$$

*where C is given by*

$$\begin{array}{ll} C & =\text{min}\left\{\sqrt{\left|\Omega \right|\left(p,p\right)}\;,\;\underset{t>0}{\text{sup}}\sum_{k=1}^{\infty }A_{k}e^{-D\left(\lambda_{k}-\lambda_{1}\right)t}\right\}\\ & \geq \text{max}\left\{A_{1},1\right\}. \end{array}$$ (12)

Before giving the proof of Theorem 2, we make a few comments about the constant *C*. First, *C* depends only on the initial distribution *p*, the domain Ω, and the escape region ∂Ω_*E*_. Note that as a result, *C* does not directly depend on the size of capture regions. Second, while *C* may be difficult to compute in general, it simplifies in certain cases. Specifically, if the initial distribution is uniform, *p*(*x*) = 1/|Ω|, then $C=\sqrt{\left|\Omega |(p,p\right)}=1$ by Eq 12. As another example, if the initial distribution is the quasi-stationary distribution, *p*(*x*) = *ϕ*_1_(*x*)/(*ϕ*_1_, 1), then the coefficients in Lemma 2 are *A*_1_ = 1 and *A*_*i*_ = 0 for *i* > 1, and it follows immediately from Eq 12 that *C* = 1.

Finally, if

$$\begin{array}{c} n/m\gg 1\;\text{and}\;nT/m\gg 1, \end{array}$$ (13)

then

$$\begin{array}{c} \begin{array}{cc} E[N] & \leq m+\frac{m}{T}(\log(\frac{n}{m}T)+1+\log\left(C\right))\\ & \approx m+\frac{m}{T}(\log(\frac{n}{m}T)+1). \end{array} \end{array}$$ (14)

Hence, in the parameter regime in Eq 13, computing *C* is somewhat superfluous since it is a subdominant term in Eq 14. Further, note that the parameter regime in Eq 13 is precisely the regime in which we expect the nonzero recharge time *τ*_*r*_ > 0 to have a nontrivial effect on E[N]. Namely, the number of particles must be much larger than the number of capture regions (*n*/*m* ≫ 1) and the recharge time must not be much smaller than the escape time (*nT*/*m* ≫ 1). This parameter regime is characterized precisely in the next section (Eq 16).

*Proof of Theorem 2*. Using Lemma 2 and the Schwarz inequality, we have

$$\begin{array}{c} S\left(t\right)\leq e^{-D\lambda_{1}t}\sum_{k=1}^{\infty }\left|A_{k}\right|\leq e^{-D\lambda_{1}t}\sqrt{\sum_{k=1}^{\infty }{\left(\phi_{k},1\right)}^{2}}\sqrt{\sum_{k=1}^{\infty }{\left(\phi_{k},p\right)}^{2}}. \end{array}$$

Since ${\left\{\phi_{k}\left(x\right)\right\}}_{k=1}^{\infty }$ are an orthonormal basis for *L*_2_(Ω), this becomes

$$\begin{array}{c} S\left(t\right)\leq \sqrt{\left|\Omega |(p,p\right)}e^{-D\lambda_{1}t}. \end{array}$$

Further, Lemma 2 gives that

$$\begin{array}{ll} S\left(t\right) & =e^{-D\lambda_{1}t}\sum_{k=1}^{\infty }A_{k}e^{-D\left(\lambda_{k}-\lambda_{1}\right)t}\\ & \leq e^{-D\lambda_{1}t}{\text{sup}}_{t>0}\sum_{k=1}^{\infty }A_{k}e^{-D\left(\lambda_{k}-\lambda_{1}\right)t}. \end{array}$$

Applying Theorem 1 completes the proof.

#### Transition between linear and logarithmic bounds

We have proven the E[N]=O(logn) as *n* → ∞ for any *T* = *τ*_*r*_/*τ*_*e*_ > 0. However, if *T* ≪ 1, then E[N] still grows linearlty in *n* for small *n*. It therefore remains to identify when E[N] transitions from linear to logarithmic growth in *n* for a given *T* > 0. In other words, when does recharge time dramatically affect E[N]?

We answer this question by determining when the logarithmic bound in Theorem 2 is better than the linear bound. Specifically, we determine the critical *n*_*c*_ such that

$$\begin{array}{c} m+\frac{m}{T}\log^{+}(C\frac{n_{c}T}{m})+\min\{Cn_{c},\frac{m}{T}\}=hn_{c}. \end{array}$$

Observe that if *Cn*_*c*_ ≤ *m*/*T*, then the linear bound is tighter than the log bound. Hence, we seek the unique *n*_*c*_ > *m*/(*TC*) such that

$$\begin{array}{c} m+\frac{m}{T}\log(C\frac{n_{c}T}{m})+\frac{m}{T}=hn_{c}. \end{array}$$ (15)

The solutions to this transcendental equation can be expressed in terms of the so-called Lambert *W* function [15]. Specifically, it is straightforward to check that the solution to Eq 15 satisfying *n*_*c*_ > *m*/(*TC*) is

$$\begin{array}{c} n_{c}=-\frac{m}{Th}W_{-1}(-\frac{h}{C}e^{-T-1}), \end{array}$$ (16)

where *W*_−1_(*z*) is the lower branch of the Lambert *W* function defined by *z* = *W*_−1_(*z*)*e*^*W*_−1_(*z*)^ and *W*_−1_(*z*) ≤ −1 for *z* ∈ [−*e*^−1^, 0). *W*_−1_(*z*) is a fairly standard function that is included in most modern computational software (additional details on this function can be found in S1 Text). Given some number *n* of initial particles, it is therefore straightforward to use Eq 16 to check if *n* > *n*_*c*_. If so, then the logarithmic bound in Theorem 2 is tighter than the linear bound and the recharge time significantly affects E[N].

### Analysis and applications of the upper bound

We now examine the upper bound and compare it to simulations in three domains: Ω^1D^, Ω^2D^, and Ω^3D^ (see Fig 2). The one-dimensional domain is the interval,

$$\begin{array}{c} \Omega^{\text{1D}}=\left[0,L_{x}^{\text{1D}}\right], \end{array}$$

with an escape region at *x* = 0 and a capture region at $x=L_{x}^{\text{1D}}$. The two-dimensional domain is the rectangle,

$$\begin{array}{c} \Omega^{\text{2D}}=\left[0,L_{x}^{\text{2D}}\right]\times \left[0,L_{y}^{\text{2D}}\right], \end{array}$$

with escape regions along *x* = 0 and $x=L_{x}^{\text{2D}}$, capture regions along *y* = 0, and reflecting boundaries for the remainder of the boundary. Lastly, the three-dimensional domain is the cylinder,

$$\begin{array}{c} \Omega^{\text{3D}}=\{\left(x,y,z\right)\;|\;x^{2}+y^{2}<{\left(R^{\text{3D}}\right)}^{2},0<z<L_{z}^{\text{3D}}\}, \end{array}$$

with escape regions at *x*^2^ + *y*^2^ = (*R*^3D^)^2^, capture regions located on *z* = 0, and reflecting boundaries for the remainder of the boundary. Unless otherwise specified, all particles are initially located at the gray triangles in each domain in Fig 2.

#### Linear vs. logarithmic growth

**Fig 3:**
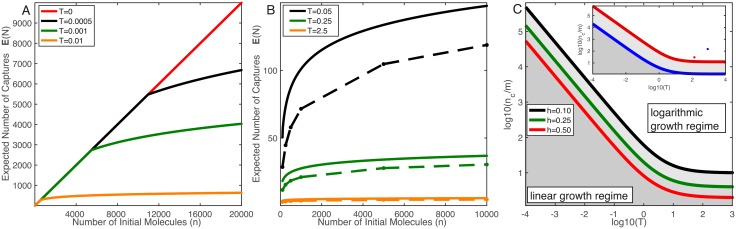
The number of captured particles depends logarithmically on the number of particles released. A: Upper bound comparisons for *T* = 0 and *T* > 0, where *T* = *τ*_*r*_/*τ*_*e*_ is the relative recharge time (non-dimensional). B: Comparison of the upper bound (solid) to simulations (dots connected with dashed lines) in Ω^1D^ with for different values of *T*. This figure suggests that the logarithmic growth found in the upper bound is observed in simulations. C: Plot of critical ratio *n*_*c*_/*m* in Eq 16 for various values of *h*. These curves split the (*T*, *n*_*c*_)-plane into two parts: parameter values that lie above the line fall into the logarithmic growth, while those that lie below it will fall into the linear growth (shaded regions). Inset: Plot of *n*_*c*_/*m* for Ω^3D^ (*C* = 1.602) with *h* = 0.85 (neuronal synapse; blue) and *h* = 0.08 (predator-prey; red); see text for more detail. The dots represent the points estimated from parameter values found in Table 2. All graphs use the parameters: *D* = 1(*μ*m)^2^/ms and $L_{x}^{\text{1D}}=1\;\mu \text{m}$.

Recalling Eq 1, if the capture regions recharge instantly (*T* = *τ*_*r*_/*τ*_*e*_ = 0), then the expected number of captured particles grows linearly in the number of initial particles. However, we found that for all *T* > 0, the linear growth can only hold for *n* < *n*_*c*_, where *n*_*c*_ is determined by Eq 16. This point is illustrated in Fig 3A, where the upper bound of Theorem 2 for Ω^1D^ is plotted for different values of *T*. This figure shows that as *T* increases, the upper bound branches off of the linear instant recharge case (red line) at smaller values of *n*.

In Fig 3B, we plot Monte Carlo estimates of E[N] and find that E[N] does indeed grow logarithmically in *n*. That is, while the theorems in the previous section provide logarithmic upper bounds for E[N], the actual expected number of captured particles does not grow sub-logarithmically. Furthermore, this figure indicates that our upper bound gets sharper as *T* gets larger. This is confirmed by calculating the percent error (figure not shown).

In Fig 3C, we plot the critical ratio, *n*_*c*_/*m* from Eq 16 as a function of *T* for different values of *h*. If parameters lie above their corresponding *n*_*c*_/*m* curve, then E[N] grows logarithmically in *n*, and therefore recharge dramatically reduces E[N]. This result allows experimentalists and modelers to determine if they need to account for recharge in their specific system.

To illustrate, we now consider two specific examples. First, we model the diffusion of neurotransmitters in a neuronal synapse containing only N-methyl-D-aspartate (NMDA) receptors with the cylindrical domain Ω^3D^ in Fig 2C. Specifically, the reflecting boundaries make up the pre- and post-synaptic terminals, the capture regions are NMDA receptors, and the escape regions represent that neurotransmitters can diffuse out of the synaptic cleft. Second, using the same cylindrical domain, we model the predator/prey experiment in [12]. This experiment placed a single ambush predator (*Chaoborus Americanus*) in a water-filled beaker, and then released prey (*Daphnia*) in order to estimate the feeding time of the predator. Here, we consider a slightly modified setup where the prey have the opportunity to escape by reaching the sides of the domain. In both examples, all particles/prey begin in the middle of the top reflecting boundary. The parameter values for these examples are in Table 2, with additional information in Materials and Methods.

Using Eq 16, we see that recharge dramatically affects both systems. In the inset of Fig 3C, we plot the corresponding *n*_*c*_/*m* curves for these examples, as well as the specific points (denoted by dots) where these examples lie, which indeed illustrates that both of these applications are well within the logarithmic growth regime. More specifically, in the case of the synapse, the expected number of captures without recharge is 2550, while our upper bound and simulation with recharge gave values of 20.3 and 20.1 respectively. We emphasize that the spatial and temporal scales of these two applications differ by several orders of magnitude, but our theory is readily applicable to both. We therefore expect that our theory will find application in many other systems.

#### Varying the number of capture regions and initial distribution of particles

The previous section examined the effects parameters *n* and *T* have on the upper bound, and we now turn to how the number of capture regions (*m*) and the initial distribution of particles come into play. For this investigation, we consider the two dimensional domain Ω^2D^, with $L_{x}^{2D}=1$ and $L_{y}^{2D}=0.1$. The capture regions will be contained in ∂Ω_*C*_ = {(*x*, 0)|0.25 ≤ *x* ≤ 0.75}, and we will examine the cases where this space is evenly distributed between 1, 2 or 4 capture regions. By construction, the probability of hitting the union of the capture regions is fixed at *h* = 0.992 regardless of the value of *m*. As a result, with an instantaneous recharge time (*T* = 0), all of the domains would capture the same average number of molecules, namely *nh* = 1984.

**Fig 4:**
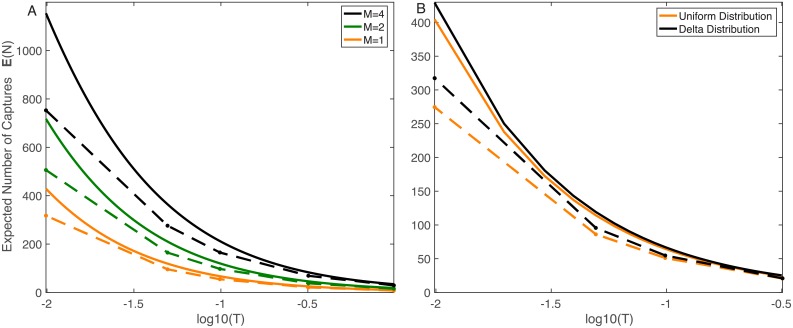
Varying number of receptors and initial conditions. Upper bound (solid) and simulations (dots connected with dashed lines). A: Comparison of expected number of captures for different numbers of capture regions, while keeping |∂Ω_*C*_|, and thus the hitting probability (*h*), constant. Even for small values of *T*, we observe large variations between the different *m* cases. B: Comparison of expected number of captures with different initial distribution of particles. For the uniform distribution, *p*(*x*, *y*) = 10 for 0 ≤ *x* ≤ 1 and 0 ≤ *y* ≤ 0.1, the constant *C* = 1 was used for the upper bound. The differences in E[N] are reduced as *T* increases. All graphs use the following parameters: domain is Ω^2D^, *n* = 2000, *D* = 1(*μ*m)^2^/ms, $L_{x}^{\text{2D}}=1\;\mu \text{m}$, $L_{y}^{\text{2D}}=0.1\;\mu \text{m}$, $\partial \Omega_{C}^{\text{2D}}=\left\{\left(x,0\right)|0.25<x<0.75\right\}$.

However, even when the relative recharge time *T* is small, we observe many fewer captures than this value, in the simulations and in the upper bound (Fig 4A). We also observe significant differences between the three different domains, with the *m* = 1 domain capturing fewer particles than the domains with two and four regions. This result is observed even though a single capture region is smaller in the *m* = 2 and *m* = 4 cases. Since |∂Ω_*C*_| is kept constant in each domain, this intuitively makes sense. However, this result is missed with an instant recharge time. Further, we note that this figure illustrates that the upper bound continues to serve as a good approximation for this two-dimensional domain, though accuracy does drop as *m* increases and *T* decreases.

We now examine how the initial distribution of particles affects the upper bound. Instead of placing all particles at a specific point in space, we assume they are uniformly distributed in Ω^2D^ at the start of the simulation. As noted previously, with this initial distribution of particles, the upper bound is much simpler to calculate, requiring only the leading eigenvalue $\lambda_{1}^{\text{2D}}$, since $C=\sqrt{\left|\Omega |(p,p\right)}=1$ by Eq 12. Fig 4B shows that this has a minor, but noticeable effect on the upper bound of E[N] and on simulation-based estimates of E[N] for smaller values of *T*. Fig 4B further shows that it has almost no effect as *T* gets larger. This can be understood by reasoning that if *T* is large, then by the time a capture region captures its first particle and recharges, the initial distribution of particles has been entirely “forgotten” by the system. On the other hand, as *T* approaches 0, we expect that the initial distribution of particles to play a bigger role in determining E[N].

#### Comparison of space dimensions

**Fig 5:**
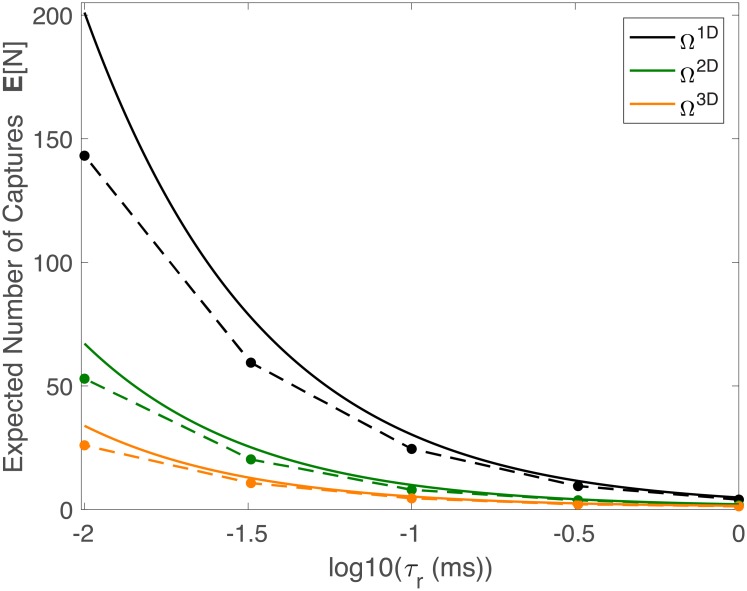
Dimension comparison. Upper bound (solid) and simulations (dots connected with dashed lines) for Ω^1D^, Ω^2D^, and Ω^3D^ with parameters chosen such that the probability of hitting a receptor in each domain is *h* = 0.5. For *τ*_*r*_ = 0, we would expect all of the lines to coincide, while the figure clearly illustrates differences in E[N] between the three domains, a characteristic predicted by our upper bound. The figure uses the following parameters: *n* = 2000, *D* = 1(*μ*m)^2^/ms, $L_{x}^{\text{1D}}=1\;\mu \text{m}$, $L_{x}^{\text{2D}}=1\;\mu \text{m}$, $L_{y}^{\text{2D}}=0.5\;\mu \text{m}$, $\partial \Omega_{E}^{\text{2D}}=\left\{\left(x,0\right)|0<x<1\right\}$, $L_{z}^{\text{3D}}=0.375\;\mu \text{m}$, *R*^3D^ = 0.5 *μ*m, ∂Ω^3D^ = {(*x*, *y*, 0)|*x*^2^ + *y*^2^ < 0.5^2^}.

We now ask the question of how the number of captured particles changes with spatial dimension. To perform this analysis, we consider the dimensional parameter *τ*_*r*_ (in *ms*) as opposed to the dimensionless parameter *T*, and domains Ω^1D^, Ω^2D^, and Ω^3D^ with parameters chosen so that *h* = 0.5 in each domain. We first note that with instant recharge (*τ*_*r*_ = 0), all of the domains capture the same number of molecules (*nh* = 500). However, if *τ*_*r*_ > 0, then the three domains capture vastly different numbers of particles (Fig 5). Indeed, Ω^1D^ captures significantly more particles than the other domains when *τ*_*r*_ is small. This result follows from the fact that even though the probability of hitting the capture region (*h*) is the same in each domain, a particle may hit a capture region while it is recharging if *τ*_*r*_ > 0. Such a particle may then diffuse away from this capture region and escape. With this set of parameters, Ω^1D^ has the largest escape time, and the result illustrated in Fig 5 follows. This result may be altered depending on the shapes and sizes of the domains, as illustrated in the next example.

**Fig 6:**
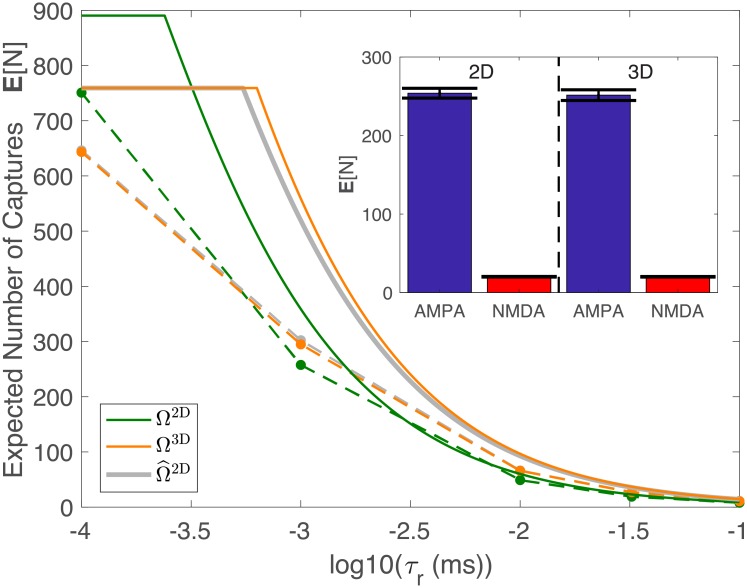
The number of capture particles in neuronal synapse (2D vs. 3D). The upper bound (solid) and simulations (dots connected with dashed lines) for Ω^2D^, Ω^3D^, and ${\hat{\Omega }}^{\text{2D}}$. Parameters were chosen such that Ω^2D^ has a smaller escape time *τ*_*e*_ and larger probability of hitting the capture region *h* than Ω^3D^. The figure illustrates that for certain values of *τ*_*r*_ it is possible for more particles to be captures in Ω^3D^. This figure used the following parameters: *n* = 1000, *D* = 1(*μ*m)^2^/ms, $L_{x}^{\text{2D}}=1\;\mu \text{m}$, $L_{y}^{\text{2D}}=0.25\;\mu \text{m}$, ∂Ω^2D^ = {(*x*, 0)|0 < *x* < 1}, $L_{z}^{\text{3D}}=0.25\;\mu \text{m}$, *R*^3D^ = 1 *μ*m, ∂Ω^3D^ = {(*x*, *y*, 0)|*x*^2^ + *y*^2^ < 0.25^2^}. The following parameters were then adjusted for ${\hat{\Omega }}^{\text{2D}}$ so that it would capture approximately the same number of particles as Ω^3D^: $L_{x}^{\text{2D}}=1.3064\;\mu \text{m}$, $L_{y}^{\text{2D}}=0.18\;\mu \text{m}$, and ∂Ω^2D^ = {(*x*, 0)|0.612 < *x* < 0.694}. Inset: Estimates of the expected value (bar) and standard deviation (line) of the number of molecules captures from simulations in the approximate synaptic cleft in two- and three-dimensions. The parameters of Ω^3D^ can be found in Table 2. Using these values, the parameters for Ω^2D^ were calculated using Algorithm 1, and were found to be $L_{x}^{\text{2D}}=0.196\;\mu \text{m}$ and $L_{y}^{\text{2D}}=0.0405$. The receptors in Ω^2D^ were uniformly distributed along {(*x*, 0)|0 < *x* < 0.196} and had radius 0.00034 *μ*m.

To further examine the effects of escape time on E[N], we now compare the number of captured particles in Ω^2D^ and Ω^3D^, where the sizes of Ω^2D^ and Ω^3D^ are chosen so that Ω^2D^ has a smaller escape time *τ*_*e*_ and larger probability *h* of hitting the capture region than Ω^3D^. With these constraints, it follows from Eq 1 that on average Ω^2D^ will capture more particles than Ω^3D^ if *τ*_*r*_ = 0. Interestingly, the upper bound in Theorem 2 suggests that Ω^3D^ may actually capture more particles than Ω^2D^ if *τ*_*r*_ is sufficiently large (see orange and green curves in Fig 6). This prediction is verified in simulations (see dashed curves in Fig 6). As in Fig 5, this counterintuitive result can be understood in terms of the smaller escape time of Ω^2D^ compared to Ω^3D^.

#### Three- and two-dimensional synapses

The previous section illustrates the difficulty in approximating a three-dimensional domain with a two-dimensional domain when the capture regions have non-instant recharge times. This type of approximation is common in computational neuroscience [16, 17]. Fig 5 suggests that it is insufficient to simply account for the probability of hitting a capture region. Further, Figs 5 and 6 suggest that the escape time *τ*_*e*_ is largely responsible for the differences in E[N] between the domains. Using this insight from our upper bound, we conclude that an accurate two-dimensional approximation must at least have the same *τ*_*e*_ and *h* as the three-dimensional domain.

To test this hypothesis, we consider a three-dimensional cylinder representative of a neuronal synapse, and seek to approximate this by a two-dimensional rectangle. The goal is to choose the parameters of the two-dimensional rectangle so that the expected number of captured molecules is the same in both domains. To choose the dimensions of our rectangle, we follow the steps outlined in Algorithm 1.

**Algorithm 1**

(1) Choose *L*_*x*_ such that $\lambda_{1}^{\text{2D}}=\lambda_{1}^{\text{3D}}$.

(2) Choose the size of the receptors in the 2D such that they make up the same proportion of boundary $\left(\text{i.e.}\;\frac{|\partial \Omega_{c}^{\text{2D}}|}{|\partial \Omega^{\text{2D}}|}=\frac{|\partial \Omega_{c}^{\text{3D}}|}{|\partial \Omega^{\text{3D}}|}\right)$.

(3) Choose *L*_*y*_ such that *h*^2D^ ≈ *h*^3D^.

We first apply this algorithm to adjust parameters for Ω^2D^ used in Fig 6 to match the results from Ω^3D^. As Fig 6 (gray line) illustrates, the algorithm produces parameter values that result in very similar upper bounds for both domains. Likewise, the simulations from the two domains are almost indistinguishable.

We now extend this concept to approximating a three-dimensional neuronal synapse, with the parameters found in Table 2. Specifically, we consider two synapses, one containing only the slow recharging NMDA receptors (*m* = 20), and another with the fast recharging AMPA receptors (*m* = 200). Using Algorithm 1, we chose parameters for Ω^2D^ so that Ω^2D^ and Ω^3D^ yielded similar values for E[N] (Fig 6 (Inset); compare bars in the left and right sides). This figure also illustrates, similar to our earlier results, that the logarithmic growth predicted by our upper bound is relevant for realistic scenarios. Specifically, while a vesicle releases approximately 10^3^ glutamate particles [8], the receptors see and bind significantly fewer, with a very pronounced difference between AMPA and NMDA receptors (red and blue bars).

## Discussion

In this paper, we considered a setup in which particles move randomly in an environment containing so-called escape regions and capture regions (traps). We have shown that if the capture regions cannot capture particles continuously but rather must recharge between captures, then the expected number of captured particles is drastically lowered compared to the case of instant recharge.

We showed this result for the case of diffusing particles, but it holds under more general assumptions on particle motion. For example, suppose each particle moves in Ω according to a Markov process with generator given by a differential operator L (this includes, for instance, the case that each particle diffuses with some deterministic drift). Then, if we can solve the PDE,

$$\frac{\partial }{\partial t}g=Lg,\;x\in \Omega ,\;t>0,$$

by separation of variables (as we did in Lemma 2 for the case of pure diffusion, L=DΔ), then we can proceed exactly as in Theorem 2. More generally, we see from Theorem 1 that the logarithmic bound on E[N] holds as long as the survival probability of each particle decays exponentially at large time.

As another generalization, we could suppose that each particles is removed from the system at a constant, spatially homogeneous rate λ_dec_ > 0. That is, suppose that in addition to (or instead of) escaping the domain, each particle has an exponentially distributed lifetime (so-called mortal walkers [18]). For instance, this would apply to second messenger proteins such as IP_3_ [19]. In this case, our results are unchanged once we replace our non-dimensional relative recharge time *T* = *τ*_*r*_/*τ*_*e*_ by *T* = *τ*_*r*_/*τ*_*e*_ + *τ*_*r*_ λ_dec_.

In closing, we comment on how our results relate to previous work. The so-called narrow escape problem is to calculate the mean first passage time of a diffusing particle to a small target on the reflecting boundary of a bounded domain. Though this problem dates back to Helmholtz [20] and Lord Rayleigh [21], its relevance to biological cell function has recently sparked a resurgence of interest (for example, see [22–25] and the review [26]). Mathematically, the problem amounts to a singular perturbation in a partial differential equation, and one seeks to characterize how the mean first passage time diverges as the size of the small target vanishes, and how this depends on the geometry and dimension of the spatial domain. In contrast to this previous work, our results do not require the capture or escape regions to be small. In addition, the logarithmic scaling we found for the expected number of captures is independent of the spatial dimension and geometry, and is therefore a fundamental effect of recharging boundaries.

Our study bears some resemblance to other studies of diffusion with stochastically switching boundary conditions. Such processes arose in the chemistry literature over thirty years ago [27–30] and have been studied more recently by mathematicians [31–36]. In some of these previous studies, each diffusing particle switches conformational state independently and can only be captured at the boundary in a certain conformational state. In other studies, the boundary changes state, and particles can only be captured when the boundary is in a certain state. These two scenarios are equivalent for a single particle. For multiple particles, the scenarios differ because the particles are independent in the former case, whereas statistical correlations arise in the latter case since all the particles diffuse in the same random environment. However, in either case the state of the boundary is unaffected by the particles. In contrast, the boundary conditions in the present work depend on the paths of particles. Mathematically, this significantly complicates the analysis because the particles can affect each other through the boundary conditions.

We also note that the effect of recharge time has been studied recently in the context of phosphorylation reactions [37–43]. Similar in spirit to our work, the kinase and phosphatase enzymes in these studies are inactive for a transitory time following each substrate modification. However, these previous works study the dynamics of a biochemical reaction network, which is very different from the escape problem considered here.

## Supporting information

S1 Text Additional mathematical details. This file contains the proof to Lemma 2, justification for calling *τ*_*e*_ ≔ (*D*λ_1_)^−1^ the escape time, and additional details about the Lambert W Function.
(PDF)
