# Supplementary material for: Receptor recharge time drastically reduces the number of captured particles
Source: PLoS Comput Biol. 2018 Mar 1;14(3):e1006015. doi: 10.1371/journal.pcbi.1006015 (PMC5849338; doi:10.1371/journal.pcbi.1006015)
Supplement: S1 Text — This file contains the proof to Lemma 2, justification for calling τe ≔ (Dλ1)−1 the escape time, and additional details about the Lambert W Function. (PDF) [file pcbi.1006015.s001.pdf]

Receptor recharge time drastically reduces the number of captured particles

Gregory Handy, Sean D. Lawley, Alla Borisyuk

Supporting Information (S1 Text)

This file contains the proof to Lemma 2, justification for calling $\tau_e := (D\lambda_1)^{-1}$ the escape time, and additional details about the Lambert W Function.

Proof of Lemma 2

Let $X(t) \in \overline{\Omega}$ denote the position at time $t \geq 0$ of a particle diffusing in $\overline{\Omega}$ with reflecting boundary conditions on $\partial\Omega$ and diffusivity $D > 0$. Let $g(x,t)$ denote the probability that the particle has not reached $\partial\Omega_E$ by time $t \geq 0$ given that it starting at $x \in \overline{\Omega}$. Precisely, define the stopping time

$$t_e := \inf\{t > 0 : X(t) \in \partial\Omega_E\}, \tag{17}$$

and let

$$g(x,t) := \mathbb{P}(t_e > t \,|\, X(0) = x).$$

It is known that $g(x,t)$ satisfies the Kolmogorov backward equation [1]

$$\begin{aligned}
\tfrac{\partial}{\partial t} g &= D\Delta g, && x \in \Omega, t > 0, && (18)\\
g &= 0, && x \in \partial\Omega_E, t > 0, && (19)\\
\tfrac{\partial}{\partial \sigma} g &= 0, && x \in \partial\Omega \backslash \partial\Omega_E, t > 0, && (20)\\
g &= 1, && x \in \Omega, t = 0. && (21)
\end{aligned}$$

By [2], there exists a set of eigenvalues and eigenfunctions as in the statement of the lemma. It is then easy to check that

$$g(x,t) = \sum_{k=1}^{\infty} (\phi_k, 1) e^{-D\lambda_k t} \phi_k(x)$$

satisfies Eqs. 18-21. If $X(0)$ is distributed according to $p(x)$, then

$$S(t) = \int_{\Omega} g(x,t) p(x)\, dx,$$

and Eq 10 follows.

Escape time.

To see why we refer to $\tau_e := (D\lambda_1)^{-1}$ as the escape time, define t_e as in Eq 17. It is known that the expected value of t_e (the so-called mean first passage time)

$$s_e(x) := \mathbb{E}[t_e \,|\, X(0) = x],$$

satisfies the elliptic problem [1]

$$\begin{aligned}
-1 &= D\Delta s_e, && x \in \Omega,\\
s_e &= 0, && x \in \partial\Omega_E,
\end{aligned}$$

$$\frac{\partial}{\partial\sigma} s_e = 0, \qquad x \in \partial\Omega \backslash \partial\Omega_E.$$

If the $X(0)$ is distributed according to its quasi-stationary distribution, $\phi_1(x)/(\phi_1, 1) \geq 0$ [3], then the mean of t_e is

$$\int_\Omega s_e(x)\phi_1(x)/(\phi_1, 1)\, dx.$$

Now, using the PDEs and boundary conditions that ϕ_1 and s_e satisfy and integrating by parts yields

$$\begin{aligned}\int_\Omega s_e(x)\phi_1(x)/(\phi_1, 1)\, dx &= -\frac{1}{\lambda_1}\int_\Omega s_e(x)\Delta\phi_1(x)/(\phi_1, 1)\, dx \\ &= -\frac{1}{\lambda_1}\int_\Omega \Delta s_e(x)\phi_1(x)/(\phi_1, 1)\, dx \\ &= \frac{1}{D\lambda_1}\int_\Omega \phi_1(x)/(\phi_1, 1)\, dx = \tau_e,\end{aligned}$$

as desired. We note that the boundary terms that appear from integrating by parts vanish since either s_e or the normal derivative of s_e is zero on the boundary.

Note on Lambert W Function

While $W_{-1}(z)$ is a fairly standard function that is included in most modern computational software, we can use recent results to obtain a more tractable description of n_c. It was recently proven [4] that $W_{-1}(z)$ satisfies

$$-W_{-1}(-e^{-u-1}) < 1 + \sqrt{2u} + u,$$

if $u > 0$. Therefore, combining this bound with Eq 16 shows that if the ratio of particles to capture regions, n/m, satisfies

$$\frac{n}{m} \geq \frac{1}{Th}\Big(1 + \sqrt{2(T + \log(C/h))} + T + \log(C/h)\Big), \tag{22}$$

then $n > n_c$ and the logarithmic bound in Theorem 2 is tighter than the linear bound. Hence, if Eq 22 is satisfied, then the recharge time significantly affects $\mathbb{E}[N]$.

Supporting References

[1] Øksendal B. Stochastic differential equations. In: Stochastic differential equations. Springer; 2003. p. 65–84.

[2] Auchmuty G. Finite energy solutions of self-adjoint elliptic mixed boundary value problems. Mathematical Methods in the Applied Sciences. 2010;33(12):1446–1462.

[3] Méléard S, Villemonais D, et al. Quasi-stationary distributions and population processes. Probability Surveys. 2012;9:340–410.

[4] Chatzigeorgiou I. Bounds on the Lambert function and their application to the outage analysis of user cooperation. IEEE Communications Letters. 2013;17(8):1505–1508.
